# The role of the polybromo-associated BAF complex in development

**DOI:** 10.1139/bcb-2024-0224

**Published:** 2024-11-14

**Authors:** JinYoung Park, Jacob G. Kirkland

**Affiliations:** a Cell Cycle and Cancer Biology Program, Oklahoma Medical Research Foundation, Oklahoma City, OK 73104, USA; b Department of Cell Biology, University of Oklahoma Health Science Center, Oklahoma City, OK 73104, USA

**Keywords:** SWI/SNF, polybromo, pBAF, chromatin, epigenetics, development

## Abstract

Chromatin is dynamically regulated during development, where structural changes affect the transcription of genes required to promote different cell types. One of the chromatin regulatory factors responsible for transcriptional regulation during development is the SWItch/Sucrose Non-Fermentable (SWI/SNF) complex, an ATP-dependent chromatin remodeling factor conserved throughout eukaryotes. The catalytic subunit of this complex, BRG1, is shared in all three SWI/SNF complexes subfamilies and is essential for developing most cell lineages. Interestingly, many human developmental diseases have correlative or causative mutations in different SWI/SNF subunits. Many polybromo-associated BAF (pBAF) complex-specific subunit genetic alterations result in developmental failures in tissue-specific ways. This observation suggests that the pBAF complex plays a vital role in development and differentiation, and studying the pBAF complex may provide an opportunity to better understand gene regulation during development. In this mini-view, we will focus on the functions of pBAF-specific subunits and their influence on the development of various cell and tissue types by regulating developmental gene expression.

## Introduction

Chromatin is a complex of proteins, including histones and DNA, that contains the genetic information needed for the diversity of gene expression required for eukaryotic life. Chromatin regulation is necessary for gene expression in an appropriate spatial and temporal manner and is essential for developing multicellular organisms. Chromatin is regulated through various mechanisms, including DNA modifications, histone variants, and histone post-translational modifications. ATP-dependent chromatin remodelers can also regulate chromatin in ways that rearrange histone distribution along the genome. The SWItch/Sucrose Non-Fermentable (SWI/SNF) complex is one of the most critical ATP-dependent chromatin remodelers. SWI/SNF complexes remodel chromatin by sliding nucleosomes or expelling histone heterodimers (Clapier et al. 2017). This chromatin alteration facilitates the access of transcriptional factors to their target genes, allowing the expression of developmentally regulated genes in the proper cell type (Alver et al. 2017). The SWI/SNF complex was first discovered in yeast (Carlson et al. 1981), but subsequent studies describe its essential role in developing multicellular organisms (Kennison and Tamkun 1988; Tamkun et al. 1992). In yeast, SWI/SNF complexes play a role in cellular metabolism and mating type switching (Stern et al. 1984). The function of the SWI/SNF complex in developmental processes was first discovered in *Drosophila*, where SWI/SNF regulates the expression of the homeobox gene cluster, which plays a critical role in Anterior-Posterior axis formation and segmentation (Tamkun et al. 1992).

**Fig. 1.**
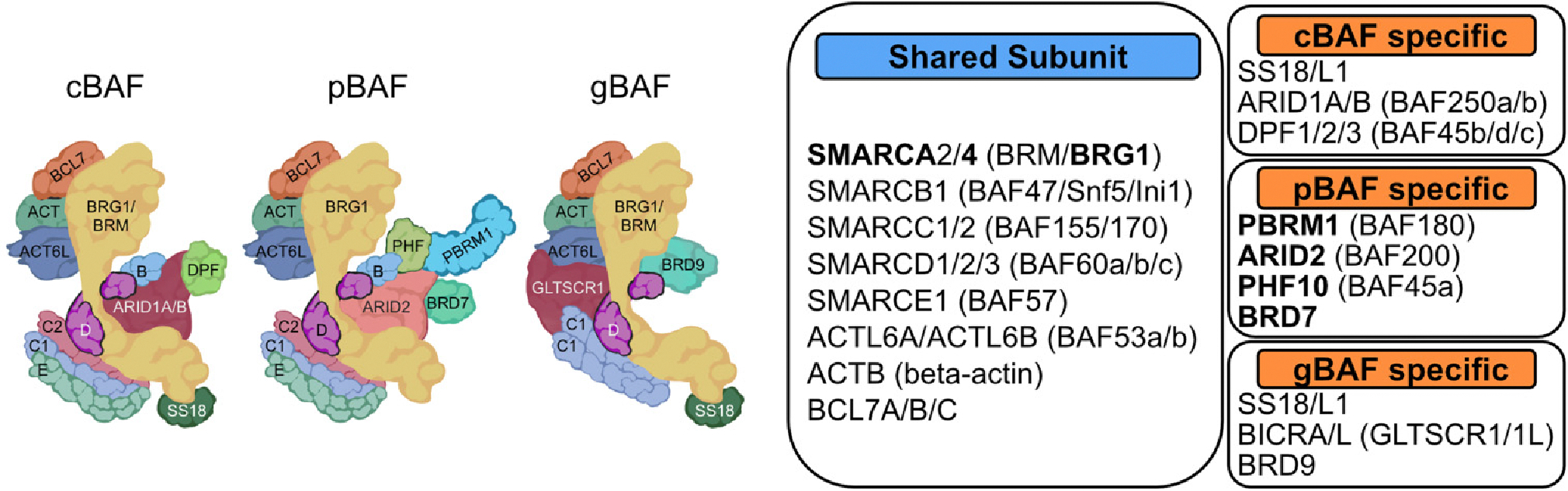
SWI/SNF complexes in mammals. (A) Schematics of the subfamilies of SWI/SNF complexes. (B) Shared subunits in SWI/SNF complexes. SWI/SNF, SWItch/Sucrose Non-Fermentable; cBAF, canonical BAF; pBAF, polybromo-associated BAF; gBAF, GLTSCR-associated BAF.

However, the function of the SWI/SNF complex during vertebrate development has yet to be fully understood. In mammals, there are three main SWI/SNF subfamilies: canonical BAF (cBAF), polybromo-associated BAF (pBAF), and non-canonical BAF (ncBAF, alternate alias gBAF) (Fig. 1A) (Mashtalir et al. 2018). Each subfamily complex contains shared subunits, but each also contains complex-specific subunits. SWI/SNF is combinatorially assembled with subfamily complex-specific subunits added midway through the assembly process (Mashtalir et al. 2018). Each SWI/SNF complex comprises 10–13 subunits encoded by 29 genes. Since each SWI/SNF complex is determined by its specific subunits, which have distinct histone and DNA binding motifs, these subunits are predicted to refine the target binding sites. Understanding the unique target sites of each SWI/SNF complex type and the role of complex-specific subunits may offer a better understanding of chromatin regulation during development. Therapeutically targeting specific complexes may also reduce toxicity compared to targeting all SWI/SNF complexes through their ATPase subunit.

Many developmental studies have focused on the ATPase subunit BRG1, contained in all three major BAF complex types, and therefore, have yet to focus on differences in the function of each complex subtype in detail. However, BRG1 deletion mutants or treatment with the ATPase inhibitor BRM014 determine a critical role for SWI/SNF complexes in chromatin regulation during development (Iurlaro et al. 2021). In mouse embryonic stem cells, inhibition of BRG1 results in the rapid loss of DNA accessibility at the binding sites of important pioneer factors such as SOX2, OCT4, and NANOG (Iurlaro et al. 2021; Schick et al. 2021). Washout of the inhibitor led to a recovery of chromatin accessibility, suggesting that this process is not heritable and, therefore, not genuinely epigenetic. These results suggest that SWI/SNF complexes play critical roles in regulating chromatin, which changes dynamically during development.

Mutations in subunits of the SWI/SNF complex are often lethal at the embryonic stage, complicating their study in later developmental processes. The cBAF complex has a unique role in neurological development (Gourisankar et al. 2024). Nevertheless, many questions about the roles of subtypes of SWI/SNF complexes during development still need to be answered. The pBAF complex contains distinct histone and other protein-binding subunits, AT-rich interactive domain-containing2 (ARID2), polybromo-1 (PBRM1), plant homeodomain finger protein 10 (PHF10), and bromodomain containing protein 7 (BRD7) (Fig. 1B) (Yuan et al. 2022; L. Wang et al. 2022). Genetic alterations of each subunit show specific mutation patterns in different cancers and other human diseases, suggesting that each subunit may have distinct functions (Hodges et al. 2016). Here, we discuss recent advancements in understanding the relationship between the pBAF complex and developmental processes, primarily focusing on the function of each pBAF-specific subunit: ARID2, PBRM1, PHF10, and BRD7.

### ARID2/BAF200

cBAF and pBAF complexes have unique AT-rich interactive domain-containing (ARID) proteins. ARID1A/B (BAF250A/B) are mutually exclusive cBAF subunits, while ARID2 (BAF200) is unique to pBAF. ARID2 has a long disordered region similar to ARID1A/B. It has an additional zinc finger domain, providing a potential mechanism for DNA targeting unique from cBAF complexes (Patsialou et al. 2005; El Hadidy and Uversky 2019).

During development, ARID2 has various functions in different tissues. However, strong phenotypes display in mesodermal lineages, such as heart (He et al. 2014), osteoblast (Xu et al. 2012), and hematopoietic stem cells (HSC) (Bluemn et al. 2021). ARID2 was first identified by interacting with serum response factor (SRF) and its co-factors, p49/STRAP and NKX2–5, where they collectively regulate cardiac gene expression (Zhang et al. 2006). Expression of ARID2 increases in cardiac tissue with age (Zhang et al. 2006). However, the consequences of this ARID2 age-dependent increase remain unknown. A null mutant of *Arid2* is embryonic lethal at E12.5–14.5 because of a heart development failure due to coronary artery malformation (He et al. 2014). Knockdown of ARID2 in pre-osteoblast cells induces a significant decrease in the expression of mesodermal lineage genes such as *BMP4* and *FGFR2* (Xu et al. 2012). In homeostasis of the hematopoietic stem cell, loss of ARID2 does not have discernable effects. However, the pBAF complex is required for cell extrinsic responses. Upon stimulation with lipopolysaccharides, cells lacking ARID2 upregulate inflammatory pathways and decrease lymphoid lineage cell proliferation (Bluemn et al. 2021).

**Fig. 2.**
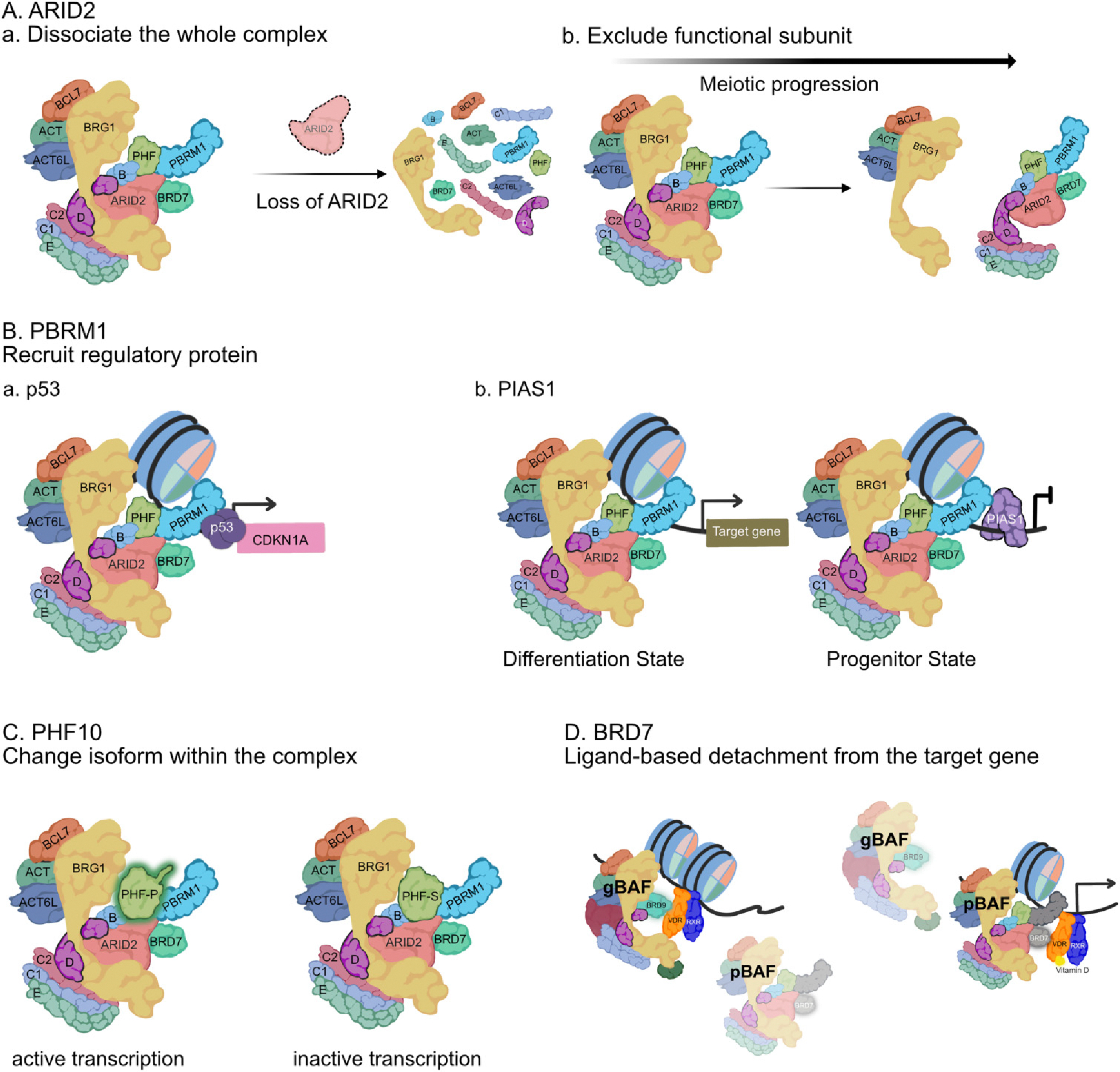
Regulatory mechanisms of pBAF-specific subunits (A) ARID2. (*a*) Disease-associated regulation mechanism of ARID2. In melanoma cells, loss of ARID2 protein leads to dissociation of pBAF-specific subunits from the complex, resulting in depletion of pBAF complexes. (*b*) Regulatory mechanism in testis. Along with meiotic progression, ARID2 generates a BRG1-free subcomplex. (B) PBRM1. (*a*) PBRM1 is necessary for expression of p21 (CDKN1A) in differentiated cells mediated by an interaction with p53. (*b*) Without dislocation from the target gene, changes in interacting proteins can cause pBAF to flip from a transcriptional activator to a transcriptional repressor. (C) PHF10. Different PHF10 isoforms can modulate RNA polymerase recruitment. (D) BRD7. Different ligands binding to the transcription factor VDR, determine the recruitment of different BAF complexes through interactions between the pBAF-specific subunit BRD7 or the gBAF-specific subunit BRD9. pBAF, polybromo-associated BAF; ARID, AT-rich interactive domain-containing; PBRM1, polybromo-1; PHF10, plant homeodomain finger protein 10; BRD7, bromodomain containing protein 7.

Coffin-Siris syndrome is an intellectual disability syndrome. Patients commonly have mutations in BAF subunits, which are thought to be causative of the syndrome. ARID1B is the most frequently mutated BAF subunit in Coffin-Siris syndrome (Santen et al. 2012; Bramswig et al. 2017). However, ARID2 mutants also affect brain development in some patients with intellectual defects, such as the NicolaidesBaraitser syndrome and the Coffin-Siris syndrome-like phenotype (Shang et al. 2015; Van Paemel et al. 2017). Patients with a mutation in ARID2 show similar but weaker phenotypes to Coffin-Siris syndrome, suggesting that ARID2 works cooperatively with cBAF in brain development or shares target sites with cBAF but regulates those independent of cBAF. The possibility that cBAF and pBAF may share functions and genomic localization has been studied through genomics studies in melanoma cells (Carcamo et al. 2022). When ARID2 is knocked out, the other pBAF-specific subunits no longer associate with the complex, leading to the loss of the pBAF complexes (Fig. 2Aa) (Carcamo et al. 2022). The loss of pBAF complexes causes the redistribution of the other BAF complexes in melanoma cells. Upon pBAF loss, cBAF and/or ncBAF binding to regions previously shared with pBAF is reduced while cBAF and/or ncBAF binding to pBAF-independent sites increases, suggesting contexts where pBAF helps target other BAF complexes to the genome. This cascade of events in ARID2 deficient cells leads to melanoma cells becoming metastatic. Because this study mapped BAF complexes using the subunit SS18 found in both cBAF and ncBAF, it is unclear if the cooperative relationship with pBAF extends to ncBAF or is primarily a function of the more abundant cBAF.

ARID2 is expressed throughout the body but is most highly expressed in the testis (Zhang et al. 2006). ARID2-containing pBAF complex interacts with kinetochore-associated Pololike kinase 1 (PLK1) and regulates spindle assembly and chromosome segregation. As a result, conditional knocking out ARID2 in spermatogonia leads to a developmental arrest in late meiosis I, where cells are deficient in spindle assembly (Menon et al. 2021). Similarly, another conditional knocking out ARID2 in spermatogonia leads to defects in chromosome spindle association at later stages of meiosis I and meiosis II without defects in DNA repair (de Castro et al. 2022). In addition to the canonical pBAF complex, ARID2 forms a BRG1-independent pBAF complex that does not contain a functional ATPase (Fig. 2Ab). The BRG1-independent ARID2 complex contains PBRM1. However, it is unknown if the other pBAF-specific subunits, PHF10 and BRD7, are a part of this complex, suggesting that the meiosis ATPasefree version of pBAF is assembled differently. The BRG1independent ARID2/PBRM1 complex is also found in mitotic cells but functions differently from meiotic cells. In mitotic cells, the BRG1-independent ARID2/PBRM1 complex is necessary for DNA repair and has the ability to interact with RAD51 (de Castro et al. 2017). The specific function of this alternate ARID2/PBRM1-containing complex is unknown. However, here we propose several models that will require further testing: (1) removal or lack of incorporation of the ATPase subunits acts to inhibit full pBAF complexes by sterically blocking pBAF binding sites, or (2) it alters the organization of the genome without remodeling nucleosomes. It is unclear what mediates the formation of BRG1-independent ARID2/PBRM1 complexes and whether these exist outside of meiosis.

### PBRM1/BAF180

PBRM1 was the original defining subunit that gave the pBAF complex its name (polybromo-associated BAF) (Xue et al. 2000). PBRM1 contains the most histone-binding motifs of any BAF subunit: six bromodomains (BDs) that bind acetyl groups, two bromo-adjacent homology (BAH) domains involved in protein–protein interaction, and one HMG domain that binds DNA (Xue et al. 2000). The bromodomains of PBRM1 interact not only with acetylated histones but also with other acetylated proteins (Karki et al. 2020) and RNA (De Silva et al. 2023). Similar to ARID2, the *Pbrm1* knockout mouse is embryonic lethal between E11.5 and E15.5 due to coronary artery defect and failure in heart chamber maturation (Wang et al. 2004; Huang et al. 2008). The regulation of heart development by PBRM1 involves retinoic acid-dependent gene activation (Wang et al. 2004).

PBRM1 has a high frequency of mutation or deletion in clear cell renal cell carcinoma (ccRCC). PBRM1 alterations are also observed in several other cancers, including non-small cell lung cancer, cholangiocarcinoma, and breast cancer at lower levels than ccRCC (Hodges et al. 2016). PBRM1 regulates the hypoxic inducible factor (HIF) pathway in kidney cells, and HIF dysregulation is a hallmark of ccRCC (Chowdhury et al. 2016).

PBRM1 binds on the promoter of *p21* in mouse embryonic fibroblasts (MEFs) and HSCs. Depletion of PBRM1 in MEFs and HSCs increases *p21* transcription and active histone marks such as H3K27ac, H3K9ac, and H3K4me1 at the *p21* promotor (Lee et al. 2016). Similarly, PBRM1-deficiency in CD8 + T cell progenitors leads to upregulation of p21 (Pan et al. 2018; Kharel et al. 2023), resulting in T cell exhaustion suggesting a repressive role of p21 by PBRM1. However, this role is not universal. Returning to ccRCC, PBRM1 deletion or mutation leads to the opposite, decreased p21 expression and a loss of PBRM1 binding to acetylated p53 in the case of PBRM1 mutation (Fig. 2Ba). In breast cancer, PBRM1 is necessary for p21 expression, regulating cell cycle arrest, and functioning as a tumor suppressor (Xia et al. 2008).

The difference in the function of PBRM1 between cells in the progenitor state, cells undergoing differentiation, or different terminally differentiated cell types may derive from interactions with other proteins. In human skin epithelial progenitor cells, PBRM1 predominantly represses terminal differentiation to sustain the progenitor’s regenerative potential (Ho et al. 2024). However, in the differentiation state, PBRM1 functions as an activator of differentiation genes. In both cases, PBRM1 localizes to the same genomic regions but switches its function depending on binding partners. PBRM1 functions as a repressor when interacting with the E3 SUMO ligase PIAS1. However, PIAS1 chromatin binding is greatly diminished during differentiation, and PBRM1 loses its repressive activity and switches to an activator. While SUMOylation is involved in this process, it is unclear if PBRM1 is being SUMOylated or if other proteins interacting or co-localizing with PBRM1 are SUMO targets (Fig. 2Bb).

PBRM1 has roles outside of gene regulation, such as maintaining genomic integrity in the context of DNA damage response (Chabanon et al. 2021) and chromosome cohesion (Brownlee et al. 2014) and chromosome segregation (Karki et al. 2021), where PBRM1 binds to methylated microtubules via BAH domains. However, transcriptional and nont-ranscriptional processes can also be coupled. PBRM1 is required for transcriptional repression at DNA double-strand breaks (Kakarougkas et al. 2014). These results broaden the potential candidate proteins that PBRM1 can interact with beyond its function as an acetylated histone reader. It also provides a new model for how PBRM1 contributes to genome stability and maintaining correct ploidy during mitosis.

### PHF10/BAF45a

**Fig. 3.**
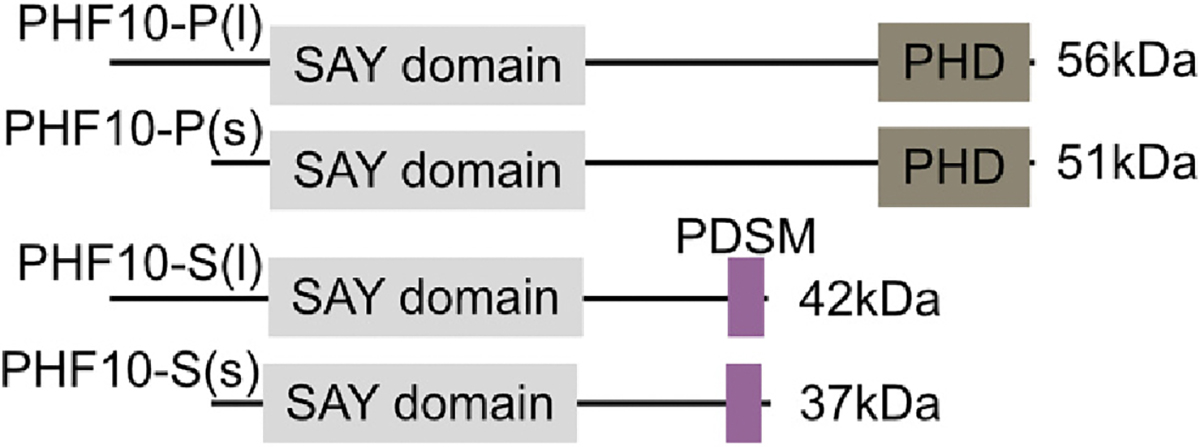
The four major human isoforms of PHF10. The -P versions contain dual PHD domains, while the -S versions contain PDSM domains. Both -P and -S classes have long and short isoforms based on N-terminal inclusion. PDSM, phosphorylation-dependent SUMO1-conjugating motif.

PHF10 is a pBAF-specific subunit, while cBAF complexes contain one of the orthogonal proteins DPF1/2/3 (Fig. 1B). PHF10 is an additional reader subunit in the pBAF complex through multiple domains. The PHF10 gene encodes for at least four different PHF10 isoforms in mice and humans (Fig. 3). All isoforms contain the conserved SAY (supporter of the activation of yellow) domains required for transcriptional activation in flies (Shidlovskii et al. 2005). SAY domains are essential for the maintenance and proliferation of neural progenitors in mammals (Lessard et al. 2007). These four isoforms fall into two major classes. The first class (PHF10-P) contains a C-terminal double PHD finger (DPF) domain that interacts with histone tails and may help target pBAF complexes to certain chromatin domains. The second class has an early termination site with the consensus sequence for phosphorylation-dependent SUMO1-conjugating motif (PDSM) at the C-terminal end (PHF10-S). Both forms can also have long (l) and short (s) forms, with the shorter forms resulting from alternative promoters and transcriptional and translational start sites. The short protein forms of PHF10-P and PHF10-S lack an N-terminal unstructured region compared to the long forms. Sizes of PHF10 isoforms vary between 37 KDa and 56 KDa in humans (Vorobyeva et al. 2009). Depending on the C-terminal domain, PHD containing PHF10 (PHF10-P) and PDSM containing PHF10 (PHF10-S) have different effects on the transcription level of genes and the recruitment of RNA polymerase II when they bind a promoter (Brechalov et al. 2014). Therefore, which isoforms are expressed in a specific cell type or which isoform is in an individual pBAF complex may influence gene expression patterns and cell lineage fates (Fig. 2C). However, since both classes of PHF10s are ubiquitously expressed in various cell types, the study of the different functions of different isoforms is more complex. A recent study further determined that PHF10-S forms begin to express when the cell lines differentiate along the neuronal and muscle lineages, whereas the expression of both PHF10-P isoforms is downregulated during this process (Bayramova et al. 2024). This isoform expression switching is most evident in the brain and heart, where the PHF10-S dominates.

Like other pBAF-specific subunits, knocking out PHF10 in mouse models is lethal in the perinatal stage of development (Krasteva et al. 2017). PHF10 is essential for maintaining hematopoietic stem and progenitor cells (HPSC), especially for long-term HPSC. Conditional knocking out *Phf10* mainly affects myeloid lineage cell differentiation, including myeloid progenitors, monocytes, and granulocytes. pBAF complexes are expressed in myelocyte progenitor cells and are repressed during differentiation, becoming very low in the terminally differentiated neutrophils. During myeloid differentiation, there is a switch from the PHF10-P isoform to the PHF10-S isoform in terminally differentiated neutrophils (Viryasova et al. 2019). PHF10-P forms are important for initiating the transcription of myeloid-specific differentiation genes. However, this is not universal, as the directed recruitment of PHF10-P containing pBAF complexes fails to evict polycomb repressive complexes and activate transcription of a developmentally regulated gene in mouse embryonic stem cells (Bergwell et al. 2024).

PHF10 has a half-life of 12 hours or less, making it one of the least stable BAF subunits. PHF10 protein is targeted for proteasomal degradation by the ubiquitin E3 ligase β-TrCP (Tatarskiy et al. 2017). This rapid turnover suggests the cell may regulate the pBAF complex through PHF10. Rapid protein turnover is consistent with more efficient isoform switching, rapidly changing pBAF functions, and differentially affecting pol-II-based transcription. The high stability of the other pBAF subunits with low turnover rates supports a PHF10 isoform-based rapid modularity model.

The function of PHF10 is challenging to study for several reasons, and new tools and directed approaches are required. First, an individual pBAF complex can only incorporate one PHF10 molecule and, therefore, only one PHF10 isoform. Second, multiple isoforms exist in the same cell type and likely in the same individual cell. Third, commonly used commercial antibodies seemingly recognize all major isoforms. Finally, multiple post-translational modifications, such as phosphorylation and SUMOylation, also regulate PHF10.

### BRD7

BRD7, like PBRM1, functions as a reader by binding acetyl groups on proteins (Kaeser et al. 2008) such as histone tails (Peng et al. 2006). A null mutant mouse model shows delayed limb development and growth retardation as early as E12.5, and all null mutant mouse embryos die by E16.5. Another *Brd7*-knockout mouse model shows defects in spermatogenesis and male infertility, but knockout mice lived to adulthood. Depletion of BRD7 arrests spermatogenesis in the postmeiotic stage, condensing spermatids. It also displays increased apoptosis and DNA damage with disrupted DNA damage response (Wang et al. 2016). The discrepancy in the viability of the *Brd7* null mutant may result from the difference in genetic background or generation of hypomorphic protein from the deletion strategy.

BRD7 is involved in several lineages of cell differentiation, such as osteogenesis (Sinha et al. 2020), hemopoiesis (Huang et al. 2024), and myogenesis (Li et al. 2022). Knockdown of BRD7 is sufficient to decrease the phosphorylation on SMAD, leading to decreased BMP signaling in mesenchymal stromal cells. These results were also shown in ARID2 and PBRM1 knockdown, suggesting that osteogenic genes are regulated by the pBAF complexes rather than individual subunits (Sinha et al. 2020).

Differentiation of naive CD8 + T cells into functional short-lived effector cells requires BRD7. The expression of BRD7 significantly increases in T cells following infection with the influenza virus. During the differentiation of effector cells, BRD7 causes changes in several genes involved, most notably binding to the promoter of *Tbx21*, which encodes T-bet, leading to a significant increase in DNA accessibility and mRNA expression without changes in histone modification (Huang et al. 2024).

TNF-ɑ decreases the expression of BRD7 in airway smooth muscle cells (ASMCs) with increased sensitivity of BRD7’s expression (Li et al. 2022). A reduced level of BRD7 enhances the pro-proliferative and pro-migrative pathways in the ASMCs, negatively regulating Notch signaling with higher expression of Notch downstream genes.

Similar to PBRM1, BRD7 is necessary for p53-mediated p21 expression. However, the knockdown of BRD7 did not disrupt the interaction between the pBAF complex and p53, indicating that BRD7 does not directly bind p53 (Burrows et al. 2010).

While BRD7 has a different amino acid sequence than the gBAF-specific subunit BRD9, they both contain bromodomains (LF. Wang et al. 2022). Therefore, genome targeting by gBAF and pBAF could overlap through their respective BRDs. One example is that BRD7 and BRD9 can bind the acetylated vitamin D3 receptor (VDR). BRD9 binds vitamin D-free VDR, whereas BRD7 exclusively interacts with vitamin D-bound VDRs in pancreatic beta cells under the presence of vitamin D. Only inhibition of BRD9 moderately enhances binding pBAF complexes on VDR, suggesting that BRD9-containing gBAF and BRD7-containing pBAF complexes shares target gene profiles (Fig. 2D) (Wei et al. 2018).

## Conclusion

The components of SWI/SNFs in mammals are more diverse and complex than those in unicellular organisms or invertebrates. Mouse models and human diseases show that pBAF subunits are critical for development and tumor suppression. While SWI/SNF has long been seen as an activator, it is clear that pBAF has both activating or repressive roles dependent on the context. However, it is unclear what drives this dichotomy and how direct versus indirect effects contribute to outcomes. It is unknown how many different variants of pBAF complexes there are, but the work from testis and our limited knowledge of PHF10 isoforms suggests that there are multiple types of pBAF and that these likely have different functions and/or targets. These complex variants are likely developmentally or cell-type regulated. Our understanding of SWI/SNF as a development regulator continues to evolve, and future studies will shed light on the additional mechanisms involved in development.

## Data Availability

This manuscript does not report data.
